# Quantitative Resistance to Plant Pathogens in Pyramiding Strategies for Durable Crop Protection

**DOI:** 10.3389/fpls.2017.01838

**Published:** 2017-10-27

**Authors:** Marie-Laure Pilet-Nayel, Benoît Moury, Valérie Caffier, Josselin Montarry, Marie-Claire Kerlan, Sylvain Fournet, Charles-Eric Durel, Régine Delourme

**Affiliations:** ^1^Institute for Genetics, Environment and Plant Protection (INRA), UMR 1349, Leu Rheu, France; ^2^PISOM, UMT INRA-Terres Inovia, Le Rheu, France; ^3^Pathologie Végétale (INRA), Montfavet, France; ^4^Research Institute of Horticulture and Seeds (INRA), UMR 1345, Beaucouzé, France

**Keywords:** quantitative trait loci, major R genes, durability, resistance mechanisms, marker-assisted-selection

## Abstract

Quantitative resistance has gained interest in plant breeding for pathogen control in low-input cropping systems. Although quantitative resistance frequently has only a partial effect and is difficult to select, it is considered more durable than major resistance (R) genes. With the exponential development of molecular markers over the past 20 years, resistance QTL have been more accurately detected and better integrated into breeding strategies for resistant varieties with increased potential for durability. This review summarizes current knowledge on the genetic inheritance, molecular basis, and durability of quantitative resistance. Based on this knowledge, we discuss how strategies that combine major R genes and QTL in crops can maintain the effectiveness of plant resistance to pathogens. Combining resistance QTL with complementary modes of action appears to be an interesting strategy for breeding effective and potentially durable resistance. Combining quantitative resistance with major R genes has proven to be a valuable approach for extending the effectiveness of major genes. In the plant genomics era, improved tools and methods are becoming available to better integrate quantitative resistance into breeding strategies. Nevertheless, optimal combinations of resistance loci will still have to be identified to preserve resistance effectiveness over time for durable crop protection.

## Introduction

Plant pathogens are major limiting factors in crop production and this has led to the extensive use of chemicals to control them. Plant genetic resistance is a promising key alternative to control crop diseases and pests. However, pathogens frequently adapt to and overcome genetic resistance especially when it is determined by major genes. Thus, quantitative resistance has gained interest in recent years to address the major challenge of genetic resistance durability.

Quantitative resistance can refer to an incomplete or partial level of resistance phenotype. It can also refer to a continuous distribution between resistant and susceptible phenotypes in a progeny, most often resulting from the segregation of alleles with variable effects at several loci. In contrast, qualitative resistance refers to either a complete or high level of resistance, or to a bimodal segregation of phenotypes in a progeny, classifying individuals into two distinct categories: resistant and susceptible. Accordingly, Niks et al. (2015) distinguished qualitative or quantitative resistance, depending on whether it refers to its effect on the phenotype or mode of inheritance.

## Quantitative Disease Resistance: Inheritance, Function, Durability

Several genes usually control quantitative resistance and are associated with genomic regions or QTL (quantitative trait loci) which contribute, each with variable effect, to the phenotype of resistance to a pathogen. Quantitative resistance often confers a partial level of resistance to the plant. It does not block but only reduces pathogen multiplication, plant colonization, and/or symptom severity. However, a combination of resistance QTL can lead to total resistance in some cases, especially when QTL have strong effects (Niks et al., 2015). For example, three QTL, *rx1, rx2*, and *rx3* were found to confer a high level of resistance of tomato to *Xanthomonas campestris* (Stall et al., 2009).

Over the past 20 years, since the development of molecular markers, many resistance QTL detection experiments have been conducted in all major crop species (Kover and Caicedo, 2001; Wilfert and Schmid-Hempel, 2008). The **genetic architecture** of quantitative resistance has often been associated with a small number of detected QTL, some with major effects and others with minor effects. In recent years, the development of next generation sequencing (NGS) technologies, coupled with the use of accession panels and multi-parental populations, has accelerated the discovery of a higher number of QTL controlling quantitative resistance. Markers closely linked to QTL, especially to QTL with minor effects, and marker haplotypes describing allelic diversity at QTL were identified (Yu and Buckler, 2006; Huang and Han, 2014; Desgroux et al., 2016). Recently, Bartoli and Roux (2017) reviewed 35 genome-wide association (GWA) studies of plant resistance to pathogens. Closely linked markers to resistance QTL were reported in 34 plant–pathogen systems and candidate genes validated in *Arabidopsis thaliana*/*Pseudomonas syringae, Ralstonia solanacearum, Xanthomonas campestris*, and *Oryza sativa*/*Magnaporthe oryzae* systems.

The effects of resistance QTL are often additive. However, QTL with epistatic effects were identified (Lefebvre and Palloix, 1996; Manzanares-Dauleux et al., 2000; Calenge et al., 2005). Resistance QTL can sometimes only be detected under certain environmental conditions (soil, climate, pathogen population), or in specific genetic backgrounds or cross types. Thus, stable QTL are highly sought after for their applicability in breeding (Calenge and Durel, 2006; Ballini et al., 2008; Danan et al., 2011; Hamon et al., 2011; Goudemand et al., 2013). When several isolates are used for QTL analysis in a given mapping population, resistance QTL can be detected as either isolate-specific (i.e., detected with only one isolate) or non-specific (i.e., broad-spectrum). In several systems, quantitative resistance was found to result from the combination of broad-spectrum QTL and QTL specific to one or several isolates (Caranta et al., 1997a; Calenge et al., 2004; Rocherieux et al., 2004). Resistance QTL were also shown to be specific or non-specific to a pathogen species, and some QTL can confer resistance to multiple pathogens (Ellis et al., 2014; Wiesner-Hanks and Nelson, 2016).

While the **molecular mechanisms** underlying major R genes have been described extensively (Michelmore et al., 2013), those underlying resistance QTL are much less well-known. In their review, Poland et al. (2009) proposed several possible functions for the ***genes underlying resistance QTL***. These included host plant development or morphology, basal defense, detoxification, transduction of defense signals, or partially altered major R genes. Since then, to our knowledge, only 15 genes with partial effects underlying quantitative resistance QTL have been cloned in plants (**Table 1**) (Niks et al., 2015; French et al., 2016). Indeed, the causal genes encode proteins with roles in pathogen recognition (NB-LRR, wall-associated kinases), signal transduction (transporters), defense (lectins, prolines), and host metabolism (transferases). Some genes are altered forms of NB-LRR genes (e.g., *Pi35*) or loss-of-function susceptibility alleles (e.g., *Pi21).* Some are expressed during a specific developmental stage (e.g., *Lr34*) or in a specific environment (e.g., *Yr36*), or confer resistance when overexpressed (e.g., *Rhg1*). Positional candidate genes located in resistance QTL genomic regions were also identified in recent studies, using genomics and GWA mapping approaches (Corwin and Kliebenstein, 2017). These genes were often found to encode downstream regulatory or defense response proteins, including transcription factors influencing the response to defense hormones, defensins, pathogenesis-related proteins and secondary metabolite enzymes. ***Genes, proteins, and metabolites regulated by quantitative resistance*** in response to pathogen infection were also identified using transcriptomics and metabolomics approaches (Kushalappa et al., 2016). Resistance-related proteins and metabolites involved in antimicrobial, toxin-degrading and cell-wall enforcing activities were identified in several plant species. In potato, quantitative resistance to late blight was mainly associated with cell wall thickening due to deposition of hydroxycinnamic acid amides, flavonoids, and alkaloids (Yogendra et al., 2015). In *Arabidopsis thaliana*, a major phytoalexin, camalexin, was induced in response to clubroot infection and associated with reduced pathogen development in heterogeneous inbred family lines carrying a major resistance QTL (Lemarie et al., 2015).

**Table 1:** Cloned genes with partial effects contributing to quantitative resistance in plants.

Plant/pathogen pathosystem	Locus	Protein domain(s)	Reference
*Arabidopsis thaliana/Xanthomonas campestris*	*RKS1^∗^* *RRS1/RPS4*	Atypical kinase NB-LRR pair	Huard-Chauveau et al., 2013 Debieu et al., 2016
*Arabidopsis thaliana/Fusarium oxysporum*	*RFO1^∗^*	Wall-associated receptor-like kinase	Diener and Ausubel, 2005
Maize/*Setosphaeria turcica*	*Htn1^∗^*	Wall-associated receptor-like kinase	Hurni et al., 2015
Maize/*Setosphaeria turcica*, *Cochliobolus heterostrophus*, *Cercospora zeae-maydis*	*qMdr*_9.02_	Caffeoyl-CoA *O*-methyltransferase	Yang et al., 2017
Maize/*Sporisorium reilianum*	*qHSR1*	Wall-associated receptor-like kinase	Zuo et al., 2015
Rice/*Magnaporthe oryzae*	*Pi21^∗^*	Heavy metal-transport detoxification	Fukuoka et al., 2009
Rice/*Magnaporthe oryzae*	*Pi35*	NB-LRR	Fukuoka et al., 2014
Rice/*Tenuivirus*	*STV11*	Sulfotransferase	Wang et al., 2014
Soybean/*Heterodera glycines*	*Rhg1*	Amino acid transporter – α-SNAP protein- wound inducible protein	Cook et al., 2012; Liu et al., 2017
Soybean/*Heterodera glycines*	*Rhg4*	Serine hydroxymethyltransferase	Liu et al., 2012
Wheat/*Puccinia triticina, P. striiformis, Blumeria graminis*	*Lr34^∗^*	ABC (Adenosine triphosphate -Binding Cassette) transporter	Krattinger et al., 2009
Wheat/*Puccinia striiformis*	*Yr36^∗^*	Kinase-START	Fu et al., 2009
Wheat/*Puccinia triticina, P. striiformis, P. graminis, Blumeria graminis*	*Lr67^∗^*	Hexose transporter	Moore et al., 2015
Wheat/*Fusarium graminearum*	*Fhb1^∗^*	Pore-forming toxin-like	Rawat et al., 2016

*^∗^Cloned QTL reported as conferring broad-spectrum resistance against various isolates of a pathogen.*

Quantitative resistance is generally more durable than qualitative resistance (Parlevliet, 2002). However, cases of major genes conferring durable resistance are known. For example, the *Mlo* gene has been used to control powdery mildew in varieties of spring barley in Europe since 1979 (Acevedo-Garcia et al., 2014). In addition, several genes conferring resistance to viruses have also shown durability (i.e., *pvr2^2^, Pvr4* in pepper, *Ry* in potato) (Garcia-Arenal and McDonald, 2003). However, in general, virulent isolates rapidly overcome major R genes (Brown, 2015). The **increased durability** of quantitative resistance could be due to (i) the partial resistance effect exerting a low selection pressure on the pathogen population, (ii) a combination of contradictory selection pressures on pathogen evolution, (iii) a low probability of multiple pathogen mutations needed to overcome multiple QTL, (iv) a combination of different resistance-associated mechanisms, which together are more difficult to overcome, and (v) a combination of resistance mechanisms acting successively at different times in the pathogen life cycle or throughout plant development (Palloix et al., 2009; Mundt, 2014). Nevertheless, experimental evolutionary studies carried out by successively passaging a pathogen on plants carrying quantitative resistance, suggested that fungi and viruses can still erode away this type of resistance (Kolmer and Leonard, 1986; Lehman and Shaner, 1997; Montarry et al., 2012). Indeed, the breakdown or erosion of quantitative resistance or resistance QTL by pathogen isolates was also observed under natural conditions. Studies of these systems have provided insight into the pathogen adaptation processes involved (Cowger and Mundt, 2002; Peressotti et al., 2010; Caffier et al., 2014, 2016; Delmas et al., 2016).

## Quantitative Disease Resistance: Integration into Pyramiding Strategies for Durable Resistance

Different approaches have been proposed for adequately deploying major R genes and resistance QTL, with the aim of increasing the durability of crop resistance to pathogens. The sustainable management of available genetic resistance factors includes (i) the use of multi-line varieties or varietal mixtures carrying different R genes or QTL (Sapoukhina et al., 2013), (ii) the rotation in space or time of various R genes (Papaix et al., 2011) and (iii) the combination (i.e., pyramiding) of R genes or QTL in the same genotype (Mundt, 2014; Brown, 2015). In spite of a relative lack of data, comparative experimental and retrospective studies of these three approaches suggested that R genes may be more durable when deployed in pyramids than in varietal mixtures or rotations (Djian-Caporalino et al., 2014; Bourguet et al., 2016). However, a recent modeling approach found that rotations in space (i.e., mosaics of fields with either resistant or susceptible cultivars) were at least as efficient and durable as pyramids (Djidjou-Demasse et al., 2017).

Breeding schemes for **pyramiding major R genes** have been extensively trialed (Collard and Mackill, 2008; Ordon and Kühne, 2014) and resulted in the development of resistant varieties that are widely cultivated (Ellis et al., 2014). Strategies involving combinations of major genes, each conferring resistance to various specific isolates, were suggested to increase resistance durability (Feechan et al., 2015). However, the appearance of multi-virulent populations in different situations could compromise the effectiveness of resistance gene pyramids (i) when virulence mutations are not independent and generate limited fitness costs for the pathogen, (ii) when one or several virulence factors pre-exist in pathogen populations or (iii) when the modes of action of specific resistances are redundant (Brown, 2015). As the independent use of resistance genes in breeding programs is often unchecked, it is likely that pathogens gradually overcome resistance genes in deployed pyramids.

Breeding schemes for **pyramiding resistance QTL** were also applied to increase resistance levels in cultivated varieties, for example in barley, wheat, bean, and pepper (St. Clair, 2010). There are few reports of studies aiming to integrate disease resistance QTL in breeding strategies, in contrast to major R genes that have been widely used in plant breeding. Most focused on QTL with major effects, and very few on QTL with minor effects. Different schemes of marker-assisted-selection (MAS) have been developed to exploit and combine resistance QTL in plants, including early selection on haplotypes in F_2_ populations, marker-assisted back-crossing (MAB) and marker-assisted recurrent selection (MARS) (St. Clair, 2010). The successful MAS published to date were obtained essentially using the MAB method (Hospital, 2009). In several studies, this method was used to create near-isogenic lines (NILs) and validate the effects of previously detected resistance QTL (Lavaud et al., 2015).

The durability of QTL pyramids was rarely evaluated. Nevertheless, QTL combinations are expected to increase durability for different reasons.

Pyramiding resistance QTL showing a varying spectrum of action on pathogen strains may generate contradictory selection pressures on pathogen evolution. Le Van et al. (2013) showed that scab resistance QTL differentially selected for *Venturia inaequalis* strains co-inoculated in a mixture. Broad-spectrum QTL did not exert differential selection pressures between strains, whereas specific QTL decreased the frequency of some strains. Consequently, pyramiding QTL with different specificities or broad spectrum QTL may be expected to increase durability. Recently, the combination of two resistance QTL, which showed additive broad-spectrum effects in NILs, was suggested as a promising strategy for breeding durable resistance in rice against rice blast (Chaipanya et al., 2017).

Pyramiding resistance QTL associated with different resistance mechanisms may affect different pathogen life-history traits such as latency, infection efficiency, plant colonization, and pathogen multiplication (**Table 2**). Resistance QTL with diversified actions on the pathogen’s life-cycle were shown for yellow rust resistance in barley NILs (Richardson et al., 2006) and brown rust resistance in wheat (Azzimonti et al., 2014). QTL acting at different stages of plant development, corresponding to different stages of the pathogen infection cycle, were also identified in two wheat cultivars with durable resistance to yellow rust (Dedryver et al., 2009). In apple, the pyramiding of three resistance QTL to scab was suggested to result in more durable resistance since the QTL were shown to act at different stages of the fungal infection cycle, from as soon as it penetrated the plant to a later stage during subcutaneous growth and sporulation (Laloi et al., 2016). In pea NILs, combinations of resistance QTL individually acting on delaying symptom appearance and/or on slowing down root colonization by *Aphanomyces euteiches* had an increased action on the two pathogen life-history traits (Lavaud et al., 2016). In maize NILs, Chung et al. (2010) identified two QTL acting on different stages of the infectious cycle (penetration, colonization) of *Setosphaeria turcica*, one of which also conferred an accumulation of callose and phenolic compounds at the points of infection. In rice, NILs that pyramided four blast disease resistance QTL (*Pi21, Pi34, qBR4-2, qBR12-1*) underlying different putative functions, exhibited increased, broad-spectrum and stable levels of resistance in multiple environments (Fukuoka et al., 2015). In addition to these experimental data, theoretical studies highlighted that a combination of QTL will be more durable if the QTL affect distinct pathogen life-history traits, especially when the evolution of repressed traits is antagonistic (Bourget et al., 2015).

**Table 2:** Examples of life-history traits or phenotypes associated with resistance QTL in plants.

Life-history trait or phenotype	Plant/pathogen pathosystem	Reference
Infectivity	Barley/*Puccinia striiformis* Maize/*Setosphaeria turcica* Pea/*Aphanomyces euteiches* Pepper/*Cucumber mosaic virus* Poplar/*Melampsora larici-populina* Soybean/*Heterodera glycines*	Richardson et al., 2006 Chung et al., 2010; Hurni et al., 2015 Lavaud et al., 2016 Caranta et al., 1997b Jorge et al., 2005 Cook et al., 2012
Latency period	Barley/*Puccinia striiformis* Barley/*Puccinia hordei* Durum wheat/*Puccinia triticina*	Richardson et al., 2006; Marcel et al., 2008; Wang et al., 2010 Marone et al., 2009
Plant colonization	*Arabidopsis thaliana/Plum pox virus* *Arabidopsis thaliana/Xanthomonas campestris* Maize/*Setosphaeria turcica* Pea/*Aphanomyces euteiches* Pepper/*Cucumber mosaic virus* Pepper/*Phytophthora capsici* Potato/*Phytophthora infestans*	Pagny et al., 2012 Huard-Chauveau et al., 2013 Chung et al., 2010 Lavaud et al., 2016 Caranta et al., 2002 Lefebvre and Palloix, 1996 Danan et al., 2009
Symptom intensity and kinetics	Barley/*Puccinia striiformis* Pea/*Didymella pinodes* Pepper/potyvirusesPotato/ *Phytophthora infestans* Rice/*Magnaporthe grisea*/*oryzae* Soybean/*Heterodera glycines* Wheat/*Puccinia triticina*	Richardson et al., 2006 Prioul et al., 2004 Caranta et al., 1997b Danan et al., 2009; Sorensen et al., 2006; Villamon et al., 2005 Talukder et al., 2004; Fukuoka et al., 2009 Cook et al., 2012 Krattinger et al., 2009; Azzimonti et al., 2014
Pathogen reproduction	Apple/*Venturia inaequalis* Pepper/*Potato virus Y* Potato/*Globodera pallida* Wheat/*Septoria tritici* Wheat/*Puccinia triticina*	Calenge et al., 2004 Quenouille et al., 2014 Caromel et al., 2003; Stevanato et al., 2015 Kelm et al., 2012 Azzimonti et al., 2014
Pathogen evolution: -Differential selection among pathogen variants -Effectiveness of resistance overcome -Effective population size	Apple/*Venturia inaequalis* Pepper/*Potato virus Y* Pepper/*Potato virus Y*	Le Van et al., 2013 Quenouille et al., 2014 Tamisier et al., 2017

Breeding schemes for **pyramiding resistance QTL and major genes** were developed to increase the effectiveness of disease resistance by diversifying the putative resistance mechanisms combined (Baumgartner et al., 2015). They were also evaluated for their potential to preserve the effectiveness of resistance conferred by major genes, either by: (i) reducing the total pathogen population size, (ii) reducing the effective pathogen population size, i.e., the number of individuals that pass on their genes to the next generation, and consequently potentially increasing genetic drift, (iii) reducing the selection effects among pathogen variants or even (iv) exerting diversifying selection pressures on these variants (Quenouille et al., 2014).

In oilseed rape, major genes conferring resistance to *Leptosphaeria maculans* were shown to be rapidly overcome under field experimental conditions by recurrent selection of pathogen populations on the *Rlm6* major gene (Brun et al., 2000) and under cultivation conditions for varieties carrying the *Rlm1* or *Rlm3* genes (Rouxel et al., 2003; Zhang et al., 2016). Eight years of recurrent selection were necessary to overcome the *Rlm6* resistance introduced in a partially resistant genetic background, compared to only 3 years in a susceptible genetic background (Brun et al., 2010; Delourme et al., 2014).

In potato, resistance to the cyst nematode *Globodera pallida* conferred by a major-effect QTL (*GpaV_vrn_*) from *Solanum vernei*, which acts by masculinizing the nematode populations, was overcome after 8 years of recurrent selection in the laboratory. The speed at which the resistance was overcome depended on the genetic background into which *GpaV_vrn_* was introduced (Fournet et al., 2013), suggesting that low-effect QTL could enhance the durability of QTL *GpaV_vrn_*. In the case of resistances to *Meloidogyne* spp. root-knot nematodes, Barbary et al. (2014, 2016) also demonstrated that the effectiveness and durability of the major genes *Me1* and *Me3* depended on the genetic background into which they were introduced.

In pepper, the major *pvr2^3^* resistance allele to *Potato virus Y* (PVY) was rapidly overcome under controlled experimental conditions (Palloix et al., 2009; Quenouille et al., 2014). The combination of *pvr2^3^* with three partial resistance QTL significantly increased resistance durability under the same conditions. Durability QTL controlling the frequency of plants with *pvr2^3^*-resistance breakdown were mapped onto the pepper genome (Quenouille et al., 2014). The evolutionary mechanisms underlying the protective effect of the resistance QTL on the major gene present in the genetic background appeared to be multiple (Quenouille et al., 2013). These included: (i) the reduced ability of PVY to multiply, (ii) the increased number of mutations required for the virus to become virulent and (iii) the slowed-down selection of virulent PVY variants.

## Conclusion and Perspectives

The development of molecular markers has led to an increase in the use of disease resistance QTL in breeding. However, minor-effect QTL are still difficult to exploit and resistance can have physiological costs for the plant (Brown and Rant, 2013). The development of genomics over the last 10 years has opened up the prospects for genomic selection of quantitative resistance, which should lead to an improved consideration of minor-effect resistance QTL in breeding programs (Poland and Rutkoski, 2016). This progress has also made it possible to reduce confidence intervals and fine-map resistance QTL, so that unfavorable linkages, such as late maturity and partial resistance to *Phytophthora infestans* in potato (Muktar et al., 2015), can be broken. It has also allowed favorable pleiotropic effects of resistance loci to be identified, such as the QTL *Lr34* which confers resistance to multiple diseases in cereals (Kolmer et al., 2008; Krattinger et al., 2016), or the major locus *Xa4* which improves multiple agronomic traits by strengthening the cell wall (Hu et al., 2017). However, phenotyping methods still need to be improved, especially for evaluating resistance components which best predict field quantitative resistance. In this way, the actions of QTL on pathogen epidemics could be better described and their use in breeding optimized (Huang et al., 2009; Willocquet et al., 2017).

Quantitative resistance appears to be more durable than qualitative resistance and has the potential to preserve major-gene effects. However, there is still a need to adequately choose resistance QTL to create optimal combinations and limit QTL erosion. The choice of QTL could be based on their spectrum of action on different strains, their effect on pathogen life traits and their underlying molecular mechanisms (Niks et al., 2015; Kushalappa et al., 2016). Recently, plant resistance genes targeted by conserved essential pathogen effectors were suggested to confer more durable resistance than those targeted by non-essential effectors (Dangl, 2013; Clarke et al., 2015). Furthermore, the development of genome editing approaches will open new prospects for creating novel specificities in resistance genes which can then be combined to preserve resistance effectiveness (Andolfo et al., 2016).

Breeding strategies for quantitative resistance still require a better understanding of the genetic, ecological and agronomic determinants of pathogen adaptation involved in QTL erosion. Further knowledge is needed on the evolutionary capacity of pathogen species, which would depend on their mutation rate, reproduction mode, dispersal ability and effective population size (McDonald and Linde, 2002). Five recent studies identified bacterial and fungal genetic determinants of pathogenicity using GWA mapping (Bartoli and Roux, 2017). A genome scan approach also identified pathogen genomic regions involved in adaptation to resistance QTL (Eoche-Bosy et al., 2017a,b). Finally, in order to reduce the risks of pathogen adaptation, resistance breeding schemes should be more thoroughly integrated with deployment strategies of resistant varieties and combination strategies of diverse disease control methods (Didelot et al., 2016).

## Author Contributions

All the authors listed made substantial intellectual contribution to the work, wrote the manuscript together and approved it for publication.

## Conflict of Interest Statement

The authors declare that the research was conducted in the absence of any commercial or financial relationships that could be construed as a potential conflict of interest.
